# Supplementary material for: Splice-Junction-Based Mapping of Alternative Isoforms in the Human Proteome
Source: Cell Rep. Author manuscript; Available in PMC 2020 Jan 15. (PMC6961840; doi:10.1016/j.celrep.2019.11.026)
Supplement: 2 [file NIHMS1546469-supplement-2.zip › DF1/PXD010154/Liver/Liver_11_GSTM4_LGLDFPNGMLGSLLAQLSFPGFPSIQLPYLIDGAHK.pdf]

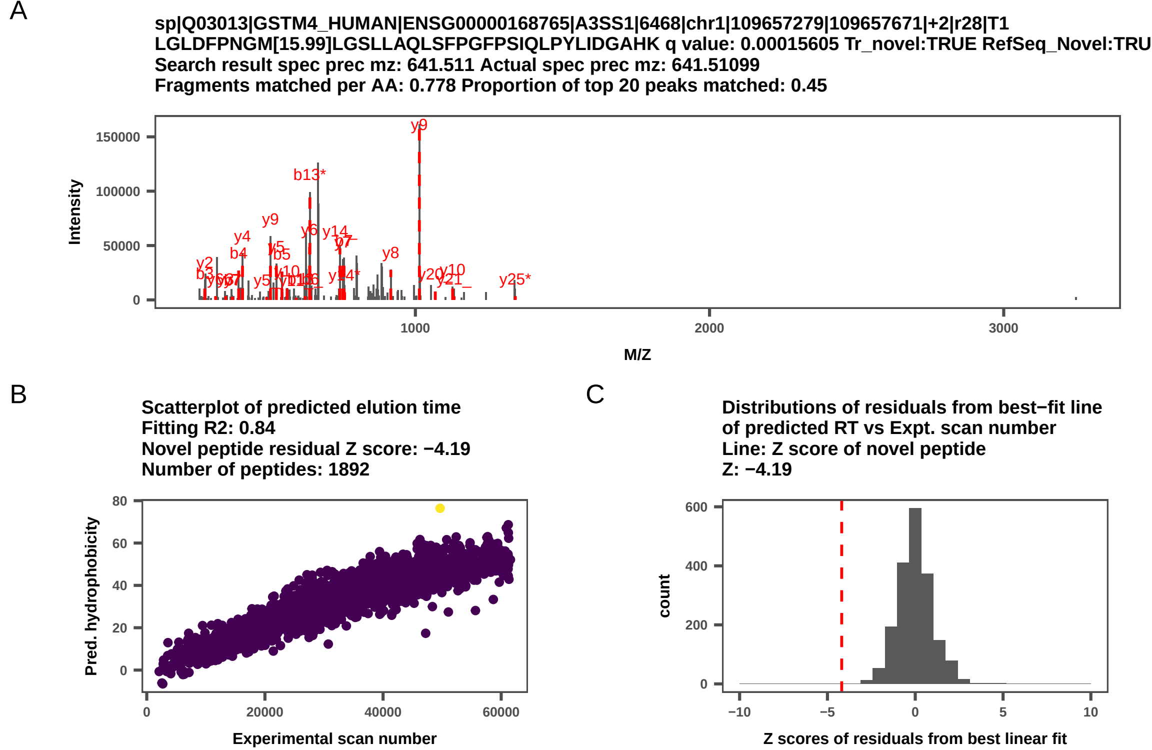
A
sp|Q03013|GSTM4_HUMAN|ENSG00000168765|A3SS1|6468|chr1|109657279|109657671|+2|r28|T1
LGLDFPNGM[15.99]LGSLLAQLSFPGFPSIQLPYLIDGAHK q value: 0.00015605 Tr_novel:TRUE RefSeq_Novel:TRU
Search result spec prec mz: 641.511 Actual spec prec mz: 641.51099
Fragments matched per AA: 0.778 Proportion of top 20 peaks matched: 0.45
Intensity
M/Z
B
Scatterplot of predicted elution time
Fitting R2: 0.84
Novel peptide residual Z score: −4.19
Number of peptides: 1892
Pred. hydrophobicity
Experimental scan number
C
Distributions of residuals from best−fit line
of predicted RT vs Expt. scan number
Line: Z score of novel peptide
Z: −4.19
count
Z scores of residuals from best linear fit
